# Addressing Gaps in Knowledge, Attitudes, and Practices in Thailand for Integrating Vaccines into a Comprehensive Dengue Management and Control Programme

**DOI:** 10.3390/ijerph23030290

**Published:** 2026-02-26

**Authors:** Darin Areechokchai, Plobkwon Ungchusak, Phatraporn Assawawongprom, Wanida Sripawadkul, Kulkanya Chokephaibulkit

**Affiliations:** 1 Office of Senior Expert Committee, Department of Disease Control, Ministry of Public Health, Nonthaburi 11000, Thailand; 2 Department of Pediatrics, Lerdsin Hospital-College of Medicine, Rangsit University, Bangkok 10500, Thailand; jui23251@hotmail.com; 3 Medical Affairs, Takeda (Thailand), Ltd., Bangkok 10330, Thailand; phatraporn.assawawongprom@takeda.com (P.A.); wanida.sripawadkul@takeda.com (W.S.); 4 Department of Pediatrics, Faculty of Medicine, Siriraj Hospital, Mahidol University, Bangkok 10700, Thailand; kulkanya.cho@mahidol.ac.th

**Keywords:** dengue, knowledge, attitude, practice, vaccine, Thailand

## Abstract

**Highlights:**

**Public health relevance—How does this work relate to a public health issue?**

- Dengue continues to place a substantial clinical and economic burden on Thailand, yet gaps in knowledge, prevention practices and vaccine awareness hinder effective dengue control.
- Understanding behavioural drivers of dengue vaccine acceptance provides critical insight into population-level barriers that limit the success of national prevention and immunisation programmes.

**Public health significance—Why is this work of significance to public health?**

- This is one of the first studies in Thailand to integrate both the KAP and COM-B frameworks to identify the behavioural, environmental and motivational determinants influencing dengue prevention and vaccine uptake.
- Findings highlight specific, measurable motivational factors, as well as safety and cost concerns, which directly affect willingness to vaccinate and can impede nationwide dengue control strategies.

**Public health implications—What are the key implications or messages for practitioners, policy makers and/or researchers in public health**

- Strengthening public communication on vaccine safety, improving accessibility through primary care, schools and workplaces, and empowering healthcare providers to address hesitancy can substantially increase vaccine willingness.
- Policymakers should integrate dengue vaccination into multi-pronged prevention efforts, combining education, vector control, and convenient delivery models, to support equitable access and build a sustainable national dengue management strategy.

**Abstract:**

Dengue remains a significant health burden in Thailand, with over 160,000 cases reported in 2023. Although two dengue vaccines are approved, uptake remains limited. This study assessed Knowledge, Attitudes, and Practices (KAP) toward dengue and behavioural drivers of vaccine willingness using the Capability, Opportunity, Motivation–Behaviour (COM-B) framework, which posits that health behaviours arise from capability (knowledge/skills), opportunity (environmental/social enablers), and motivation (beliefs/drivers). A cross-sectional online survey was conducted in September 2024 among 600 Thai adults aged 20–60 years. The questionnaire, adapted from the GEMKAP study, generated composite KAP and COM-B scores (0–100%). Willingness to vaccinate was measured on a 0–10 Juster scale, with multivariable regression identifying behavioural predictors. Of 600 respondents, 40% were male, with a median age of 40 years, and 23% were in high-dengue-burden areas. Knowledge scores were moderate (51%), and dengue prevention practices were low (40%). The proportion of respondents with high willingness to vaccinate (score 8–10) was 68%, which was positively associated with Reflective Motivation and Physical Opportunity. Hesitancy centred on vaccine side effects (29%) and cost concerns (13%). These findings suggest that despite generally favourable attitudes, vaccine uptake is hindered by safety, cost, and awareness gaps. Physician communication and the integration of vaccines into schools, workplaces, and primary care, along with education and vector control, are key for sustainable national coverage.

## 1. Introduction

Mosquito-borne infections such as dengue have increasingly become a global public health concern, especially in Southeast Asia. People living in this region account for a third of people living in dengue-endemic countries globally, driven by favourable climatic conditions, rapid urbanisation, and human mobility, which support *Aedes aegypti* transmission [1]. Particularly, Thailand is among the 30 most highly dengue-endemic countries worldwide [1].

Thailand has been dengue-endemic for over six decades, with the disease affecting all regions year-round [2]. Ten provinces (Tak, Pathum Thani, Samut Prakan, Bangkok, Chanthaburi, Trat, Phuket, Songkhla, Narathiwat, and Satun) are classified as high-risk transmission zones [3]. In 2023 alone, over 160,000 dengue cases were reported, although true incidence is likely to be up to 12 times higher due to underreporting [4]. Dengue-related deaths increased from 34 in 2022 to 147 in 2023 [5]. Beyond clinical impact, dengue imposes substantial economic and societal burdens, costing an estimated USD 440 million annually and resulting in an average of seven workdays lost per infection [4].

Given the complex interplay of biological, environmental, and socioeconomic drivers, effective dengue control requires an integrated, multisectoral approach. Thailand has implemented several national strategies, including the “3-3-1” strategy (mandating rapid case reporting, targeted mosquito elimination, and timely insecticide spraying), as well as leveraging its nationwide network of over one million Village Health Volunteers (VHVs) to support community-based prevention and surveillance [6]. Despite these longstanding efforts, dengue incidence remains high, suggesting that programmatic effectiveness may be constrained by persistent gaps in public knowledge, risk perception, and preventive behaviours.

Knowledge, Attitudes, and Practices (KAP) studies conducted in Thailand over the past three decades provide evidence of such gaps but are largely limited in scope. Community and sub-national studies, predominantly conducted in rural settings, have reported high awareness of dengue transmission pathways, but persistent misconceptions regarding treatment, prevention practices, and vector control measures. For example, a rural KAP study in northern Thailand found that while most respondents recognised mosquitoes as the dengue vector, knowledge of appropriate preventive practices and treatment options remained limited, particularly among non-agricultural and lower-education groups [7]. Other Thai studies have similarly highlighted discrepancies between knowledge and practice, with relatively positive attitudes toward dengue prevention not consistently translating into effective household-level behaviours [8].

More recently, a systematic review of dengue-related KAP studies conducted in Thailand synthesised findings across multiple community-based surveys and highlighted consistently low levels of knowledge and preventive practices despite comparatively favourable attitudes toward dengue prevention [9]. The review further noted substantial heterogeneity across studies, with many relying on geographically restricted samples or older datasets, or having a primary focus on vector control behaviours rather than vaccination awareness or behavioural drivers of vaccine uptake. Importantly, vaccine-related knowledge and attitudes were largely absent from the Thai KAP literature reviewed.

Alongside vector control, dengue vaccination represents an increasingly important preventive strategy. Two dengue vaccines have been approved by the Thai FDA: CYD-TDV in 2017 and TAK-003 in 2023 [10,11]. While vaccines are a critical solution for disease prevention, studies report that a combined intervention programme involving public health education and awareness, vector control, and vaccination strategies was cost-effective and provided the highest reduction in dengue cases compared to standalone interventions [12]. Recognising the impact of dengue and the potential benefits of dengue vaccines, the Ministry of Public Health is currently conducting a large clinical trial of TAK-003 in Nakhon Phanom province. The trial involves 35,000 children aged seven to ten to assess the vaccine’s effectiveness and its potential inclusion in the Universal Health Coverage scheme [13]. However, successful vaccine integration will depend not only on clinical effectiveness and policy decisions, but also on public understanding, acceptance, and behavioural readiness.

To our knowledge, this study is amongst the first to examine dengue-related Knowledge, Attitudes, and Practices among a broad adult population in Thailand while explicitly integrating the Capability, Opportunity, and Motivation–Behaviour (COM-B) framework to assess behavioural determinants of vaccine uptake [14]. The COM-B model links behaviour to an individual’s Capability, Opportunity, and Motivation, making it useful for understanding enablers and barriers to vaccine uptake [15]. In this study, the KAP framework assessed population-level Knowledge, Attitudes, and Practices related to dengue prevention, while COM-B provided insights into behavioural enablers and barriers to vaccine uptake. These findings can inform the development of a comprehensive dengue management plan, integrating vaccination with existing strategies in vector control, workplace health promotion, and education.

## 2. Materials and Methods

This study adapted and localised study materials from a global dengue study published in 2023, Knowledge, Attitudes and Practices toward Dengue Fever, Vector Control, and Vaccine Acceptance Among the General Population in Countries from Latin America and Asia Pacific: A Cross-Sectional Study (GEMKAP) [16]. Data for the GEMKAP study were collected in 2022 across seven countries: Argentina, Brazil, Colombia, Mexico in the Latin America region, and Indonesia, Malaysia, and Singapore in the Asia Pacific (APAC) region. It assessed the general population’s views towards dengue, including vector control, prevention behaviours and vaccination. Core study materials, including the study protocol, statistical analysis plan, data management plan, and questionnaire, were adapted for relevance to the Thai context. Modifications were made to reflect country-specific factors, including local disease burden, health system structures, and policy environment, while maintaining consistency with the original GEMKAP framework to allow for cross-country comparisons. KAP composite scores were standardised to a scale of 0 to 100%, with a higher score indicating higher levels of Knowledge, Attitudes, and Practices. COM composite scores were similarly standardised to a scale of 0–100%, with a higher score indicating higher levels. A literature review was conducted to obtain the Thailand-specific context. Additionally, to provide local context and validate the study findings, two Thai experts, an epidemiologist and an infectious disease specialist, reviewed the study materials and provided their input on the findings, which were incorporated into the manuscript to ensure the results were robust, relevant, and accurately reflected Thailand’s dengue disease landscape and management practices.

### 2.1. Study Design

This study was a cross-sectional, quantitative electronic survey conducted from 12 to 30 September 2024, to assess the KAP regarding dengue disease and vaccines among the adult population in Thailand. The study was designed to generate evidence for improving dengue prevention efforts, particularly to inform future vaccine rollout strategies. The study was conducted in accordance with the Checklist for Reporting Results of Internet E-Surveys (CHERRIES) to ensure the accuracy, validity, and reliability of the online survey methodology and data reporting [17].

### 2.2. Participants

A total of 600 Thai respondents completed the 35-question survey administered in the Thai language. The sample size was calculated to provide a ±4% margin of error at a 95% confidence level for descriptive estimates based on the full sample. Subgroup analyses are subject to wider uncertainty due to smaller stratum sample sizes.

Potential survey respondents were recruited through an existing general-purpose web-based panel via email invitations sent through Kantar Profiles’ panel mailing list. Panel members are recruited using email invitations distributed by the agency and join through double opt-in registration to validate the respondent. The study included 600 adult respondents in Thailand, which was sufficient for descriptive analysis. Eligible participants were between 20 and 60 years old, responsible for their own health decisions, and had not participated in a dengue-related survey within the past three months. Quota sampling was applied across age, income level, and geographic region to ensure sufficient representation across key demographic subgroups for descriptive analysis and statistical subgroup analysis, rather than to generate population-weighted or probability-based estimates. Quotas were guided by findings from publicly available government census data [18,19,20]. Participation was voluntary, and respondents received an incentive in the form of points that could be exchanged for prizes upon completing the survey.

### 2.3. Electronic Survey Development

The questionnaire was adapted from the GEMKAP study [16] and updated with literature findings from published dengue KAP studies conducted in Thailand to ensure relevance to the Thai context. The questionnaire covered five key themes (Supplementary File S1):

i. General knowledge and attitudes towards dengue infection;
ii. Dengue prevention;
iii. Perception of dengue vaccine;
iv. Dengue vaccination + vector control programme;
v. Education.

The questionnaire was translated and back-translated between Thai and English to ensure accuracy. Two cognitive interviews, consisting of short one-to-one pre-tests in Thai, where participants read each item and described their interpretation of the question and their answer choice, were conducted to refine, optimise, and validate the questionnaire for local comprehension, recall judgement, and response. Feedback from the cognitive interviews was incorporated before survey deployment. Additional mechanisms were incorporated into the questionnaire to minimise bias.

To minimise response bias, several design and quality control measures were implemented. For questions with long lists of options, randomisation was applied to reduce primacy bias, which is the tendency of respondents to select earlier-listed choices. To ensure data quality and prevent duplicate participation, multiple validation protocols were used in line with the CHERRIES checklist [17]. These included mandatory completion of survey fields, Internet Protocol (IP) address verification, identity authentication, and digital fingerprinting.

Additional safeguards were applied to detect and exclude inattentive or invalid responses. These included logic checks for inconsistent answers, screening for straight-lining or repetitive patterns, and reviews of nonsensical or incomprehensible free-text entries. A data cleaning process also assessed timestamp integrity to flag unusually fast completions or suspicious activity. Each participant was permitted to complete the survey only once, and any duplicate entries were removed before analysis.

### 2.4. Covariates and Outcomes

Sociodemographic variables collected included gender, age, household size, ethnicity, religion, region of residence, level of education, and household income. Other baseline characteristics, such as dengue experience, perceived risk of contracting dengue, and previous vaccination experience against dengue and COVID-19, were also collected.

The primary outcome was willingness to vaccinate against dengue, measured on a scale from 0 to 10, with responses categorised into low (0–3), moderate (4–7), and high willingness (8–10). Secondary outcomes focused on KAP regarding dengue infection, symptoms, prevention methods, and vaccines. Each survey question was assigned to a KAP subcategory, and composite scores were calculated for each subcategory and standardised on a 0 to 100% scale (Supplementary Table S1). Scores of 80–100% were classified as high, 50–79% as moderate, and 49% or below as low. These thresholds were based on established methodologies from previous KAP and dengue-related studies [21,22].

Additionally, the COM-B framework was applied to explore factors associated with respondents’ willingness to vaccinate. COM-B posits that behaviour (B) arises from three domains: Capability, Opportunity, and Motivation. Capability was divided into Physical Capability (e.g., physical skills or ability to undergo vaccination, including managing fear of needles) and Psychological Capability (e.g., knowledge and understanding of dengue transmission, prevention methods, and vaccine availability). Opportunity was divided into Physical Opportunity (e.g., environmental and structural factors, including convenient access points, scheduling, and affordability) and Social Opportunity (e.g., interpersonal and cultural influences, such as physician recommendations, government reminders, or community support). Motivation included Reflective Motivation (e.g., deliberate beliefs and evaluations, including perceived severity of dengue, trust in vaccines, and confidence in the healthcare system) and Automatic Motivation (e.g., emotional responses, habits, and incentive-driven behaviours). Survey questions were mapped to these subdomains (Supplementary Table S2) and subsequently used to inform a multivariable regression model examining associations with willingness to vaccinate (Supplementary Table S3). This evidence-based approach facilitates understanding of vaccine hesitancy by identifying gaps in individuals’ Capability, Opportunity, and Motivation that affect vaccination behaviour. Recognising the framework’s value in uncovering such insights, the COM-B framework was adopted by the World Health Organisation’s Regional Office for Europe as part of its Tailoring Immunisation Programmes (TIP) strategy [16]. Similarly to the GEMKAP study, the composite scores for COM-B measures were also calculated on a 0 to 100% scale. Scores of 80–100% were classified as high, 50–79% as moderate, and 49% or below as low. Statistical analyses were also conducted to identify factors significantly associated with higher levels of KAP and COM-B, with significance determined at *p* < 0.05.

### 2.5. Data Analysis

Descriptive analysis was used to examine sociodemographic and baseline characteristics of respondents, as well as primary and secondary outcomes, using percentages and means. Descriptive analysis provides an overview of the data, key trends, frequencies, and central tendencies within the surveyed sample. In addition, multivariable regression analyses were conducted to examine associations between behavioural factors and willingness to vaccinate. Given the quota-based sampling design, analyses were not intended to generate population-weighted estimates or probability-based inference. Subgroup analyses were conducted to identify statistically significant associations between covariates and secondary outcomes using the Wilcoxon rank-sum test, and the Holm’s method was used to control the Family-Wise Error Rate at 0.05. Subgroup comparisons are intended to highlight relative differences across groups and are subject to uncertainty driven by the corresponding subgroup sample sizes. Multivariate regression analysis was conducted using generalised linear models to identify associations between covariates and willingness to vaccinate. All statistical analyses were performed using R, version 4.3.1.

### 2.6. Ethics and Data Confidentiality

The study protocol received exemption status from the Pearl Institutional Review Board (registration number: 00007772), an independent review board fully accredited by the Association for the Accreditation of Human Research Protection Program Inc. (AAHRPP) [23,24]. Participants provided informed consent electronically, and data were handled anonymously in compliance with local privacy laws. No personal identification information was collected, stored or transferred during the survey. Data were analysed in aggregate and stored securely with permission-based access.

## 3. Results

**Figure 1:**
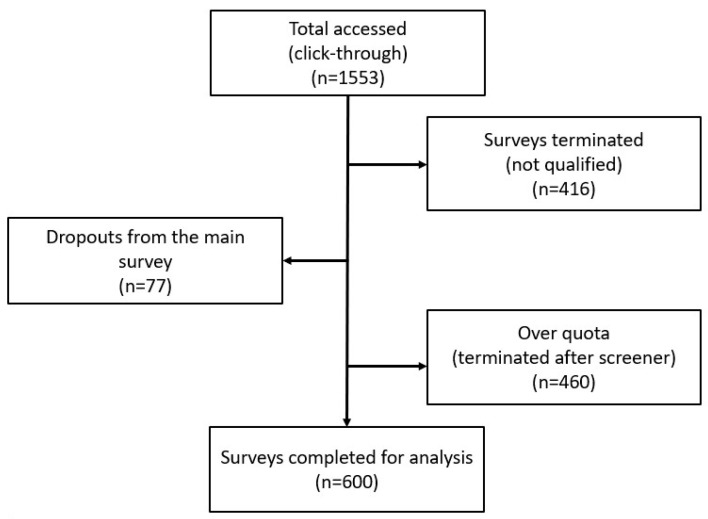
Flow chart of survey response rate.

A total of 1602 individuals accessed the screener questionnaire. Of these, 416 were disqualified for not passing the screener questions, 77 did not complete the survey, and in 460, the questionnaire was terminated due to quota overfill. A total sample size of 600 respondents was analysed for this study (Figure 1).

The study population was broadly distributed across age groups, with 23.6% aged 20–29 years, 24.7% aged 30–39 years, 24.7% aged 40–49 years, and 27.0% aged 50–60 years, and 40% of respondents were male. Most participants had attained tertiary education (71%) and reported low to medium household income (74%) (Table 1).

Out of the 600 respondents, more than half of the respondents lived in a three- to four-person household (55%), most respondents had two or fewer children (97.5%), and 64% resided in the central and northeast regions of the country. Across the respondents, 23% lived in highly endemic areas, which were defined as those with moderate to high transmission levels by the Ministry of Health (10 out of 77 provinces based on government statistics) [3]. Almost half of the respondents (43%) reported being previously infected by dengue.

### 3.1. Knowledge, Attitudes, and Practices (KAP)

**Figure 2:**
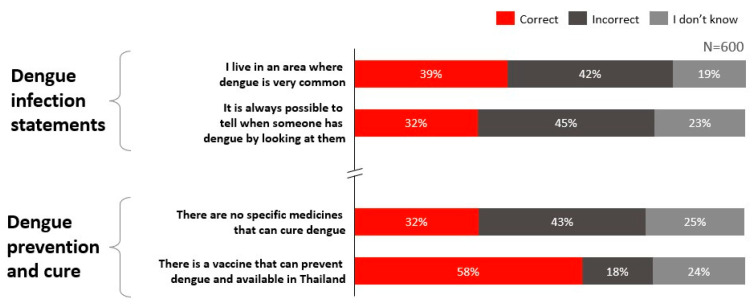
Knowledge levels regarding dengue infection and prevention in Thailand.

In Thailand, the overall Knowledge score was moderately low at 51%, indicating partial awareness of dengue transmission and prevention alongside notable gaps. Awareness of dengue endemicity and asymptomatic presentation was limited, where only 39% of respondents correctly identified whether they reside in a highly endemic area (Figure 2). 

Knowledge of dengue diagnosis was high, with 82% of Thai respondents aware of available diagnostic methods. However, knowledge related to disease risks, vaccine availability, and treatment options was comparatively lower. Only 58% of respondents were aware that dengue vaccines are available in Thailand, and only 32% correctly recognised that there is currently no specific cure for dengue.

The overall level of Attitude in Thailand was moderately low at 64%, reflecting generally favourable perceptions toward dengue prevention and vaccination. Individually implemented prevention practices, such as proper garbage disposal, draining stagnant water, and covering water containers, were perceived to be safer and more effective than community-based interventions like larval index monitoring or the sterile insect technique (SIT).

**Figure 3:**
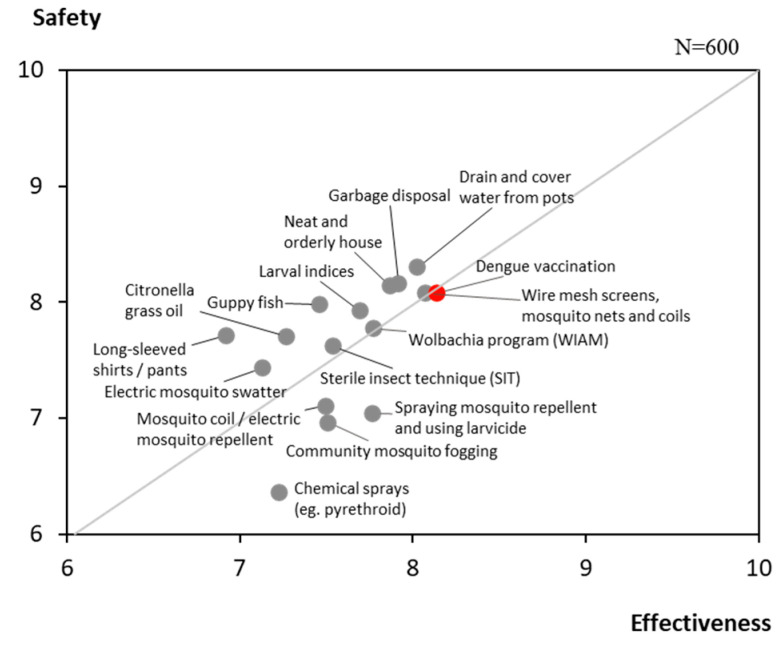
Attitudes on the safety and effectiveness of dengue prevention methods.

**Figure 4:**
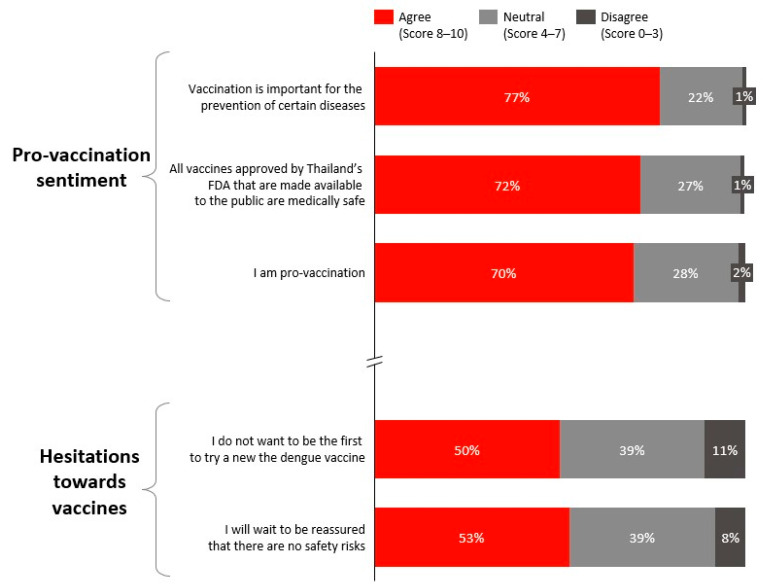
Attitudes towards vaccination.

Dengue vaccination was also viewed favourably, with respondents rating its safety and effectiveness at an average of 8.1 out of 10 (Figure 3). While 70% identified themselves as pro-vaccination, nearly half indicated a preference to wait for further reassurance on vaccine safety before proceeding with vaccination (Figure 4).

Despite moderate knowledge and generally positive attitudes, preventive Practices were comparatively lower, with respondents reporting the implementation of an average of 7.6 out of 15 recommended dengue prevention measures (Table 2). The most commonly adopted behaviours included disposing of open bodies of water (e.g., plant containers, old buckets), using wire mesh mosquito screens, and tightly covering water containers. Collectively, these findings highlight a gap between awareness, attitudes, and consistent preventive action, which is explored further in the Discussion.

### 3.2. Willingness to Vaccinate

Willingness to vaccinate against dengue among Thai respondents was generally high (Table 3). Overall, 68% expressed high willingness to vaccinate (score of 8–10 on a 10-point Juster scale), followed by 27% for moderate willingness (score of 4–7), while only 5% expressed low willingness (score of 0–3). When presented with a scenario in which a healthcare professional (HCP) recommended dengue vaccination, the proportion of respondents expressing high willingness increased to 77%.

Subgroup analysis showed that a high willingness to vaccinate against dengue (score of 8–10) was more common among Thai respondents with prior dengue infection (73%), respondents with higher perceived risk of dengue (85%), and those with positive attitudes toward vaccination (87%).

Willingness to vaccinate was also higher among individuals with higher levels of education, and those living in larger households (75%: 5–6-member households; 82%: more than 6-member households). In contrast, willingness to vaccinate did not vary significantly by gender, age group, or pregnancy status.

**Figure 5:**
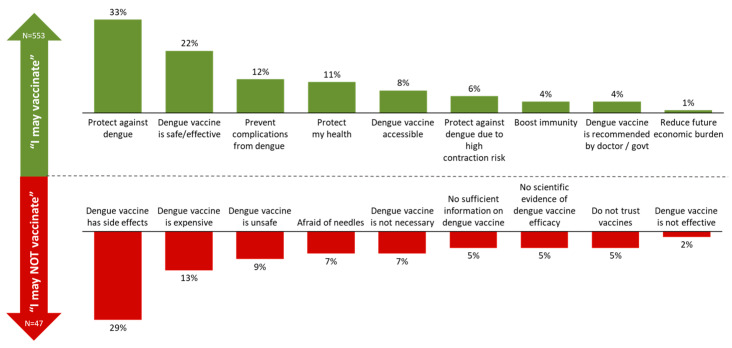
Top reasons for considering and hesitating about vaccination.

Key reasons identified for considering dengue vaccination include protection against dengue (33%) and safety and efficacy (22%), while concerns centred on potential side effects (29%) and cost (13%) (Figure 5).

### 3.3. Behaviour Lever That Can Drive Vaccine Acceptance: Capability, Opportunity, and Motivation (COM-B)

Among the COM components, Opportunity received the highest average score. In contrast, Capability scored the lowest at 57%, compared to 67% for Opportunity and 60% for Motivation (Table 4). The lower Capability score was primarily driven by gaps in both Physical and Psychological Capability, reflecting lower prevention measures practised (50%) and knowledge gaps around dengue infection, the presence of vaccine availability and the absence of a cure.

Within Opportunity, Physical Opportunity scored notably higher (78%) than Social Opportunity (66%). The higher Physical Opportunity score is reflected by the respondents’ agreement that vaccines should be launched in physically accessible places (71%) and acknowledgement that the government has ensured that access to vaccines is convenient, with clear vaccine schedules (61%).

For Motivation, Reflective Motivation (e.g., conscious beliefs and deliberate decision-making) scored higher (60%) than Automatic Motivation (e.g., emotional drivers, habits, and intrinsic motivators such as fear of illness) (38%). This indicates that willingness to vaccinate is more strongly influenced by rational considerations, such as understanding vaccine safety (66%) and recognising its importance for prevention (77%), than by emotional responses.

Regression analysis modelled that higher Reflective Motivation was strongly associated with increased willingness to vaccinate (*p* < 0.001), indicating that individuals who understand the benefits of vaccination and risks of dengue are more inclined to take preventative action. Similarly, Physical Opportunity, such as easy access to vaccines and related services, was positively associated with willingness (*p* < 0.001). In contrast, Physical Capability was negatively associated with willingness (coefficient = −1.48, *p* < 0.001). This suggests that individuals already engaged in multiple preventive practices may feel that vaccination is less necessary.

### 3.4. Influence and Health Information

**Figure 6:**
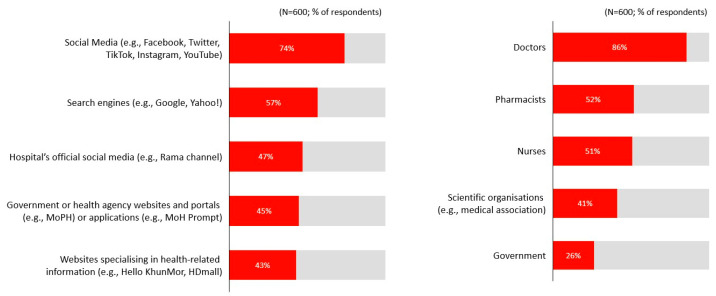
Preferred channels and trusted stakeholders.

When seeking health-related information, social media platforms such as Facebook, Twitter, and TikTok were the most preferred channels (74%). This was followed by search engines (57%) and official hospital communication channels such as the Rama channel (47%) (Figure 6). Doctors were identified as the most trusted sources of information (86%), followed by pharmacists (52%), nurses (51%), and professional medical associations (41%) (Figure 6).

**Figure 7:**
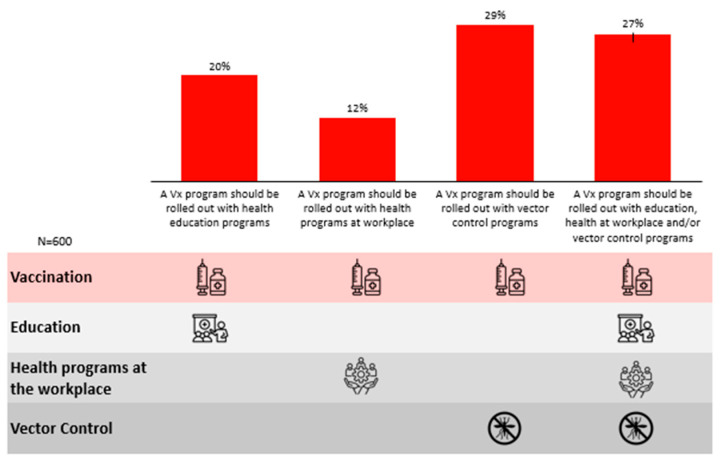
Preferred approaches towards nationwide dengue management.

Lastly, respondents expressed a preference for a multi-pronged approach towards a vaccination management programme (27%), involving vaccination, education and vector control initiatives (Figure 7).

## 4. Discussion

### 4.1. Addressing Current KAP Gaps Regarding Dengue Disease and Prevention, Including Vaccines

This cross-sectional study assesses the Knowledge, Attitudes, and Practices of Thai adults toward dengue and willingness to vaccinate. The findings highlight a persistent gap between risk awareness and preventive action. When comparing results from the GEMKAP study’s global and APAC data, Knowledge scores in Thailand averaged 51%, slightly higher than APAC (47%) and global (48%) levels. Attitude scores in Thailand (64%) were comparable to APAC (63%) and global levels (66%). Lastly, Practice scores were lower in Thailand (40%) compared to APAC (47%) and global levels (44%).

Thailand’s relatively higher Knowledge score may reflect the country’s long-standing dengue endemicity and sustained public health prioritisation, where repeated outbreaks have been accompanied by extensive public health campaigns and media attention that reinforce general disease awareness [9]. In contrast, lower Practice scores may be influenced by prevailing perceptions of dengue as a seasonal threat, historically linked to the rainy season, a period when cases typically rise due to favourable mosquito breeding conditions in Thailand [25], leading individuals to adopt preventive behaviours reactively during peak transmission periods rather than as consistent, year-round habits. Furthermore, local studies indicate that misconceptions around dengue transmission are primarily confined to rural areas and that severe disease or mortality occurs mainly in children rather than adults, which may reduce perceived personal risk among urban and working-age adults, contributing to inconsistent preventive practices [26].

Despite widespread recognition of dengue as a serious illness, a critical knowledge gap identified in this study was the misconception surrounding dengue treatment, with 68% of respondents unaware that there is no specific curative treatment. Thailand-specific studies have similarly reported beliefs that severe dengue outcomes are unlikely in otherwise healthy adults [26]. Such misunderstandings may contribute to delayed care-seeking or reliance on ineffective self-management strategies, underscoring the need for clearer public messaging on treatment realities and risk across age groups.

Likewise, while individuals expressed favourable views toward vaccination, hesitancy was evident, driven not only by cost perceptions, but also by concerns about long-term safety, which are factors commonly associated with vaccine hesitancy, including among individuals with underlying chronic conditions [27]. Thailand-specific evidence suggests that some healthy adults perceive vaccination as unnecessary unless they consider themselves at elevated risk, reinforcing a “wait-and-see” approach toward newer vaccines [27,28]. This perception may be shaped by Thailand’s national immunisation programme, which has traditionally prioritised childhood vaccination and targeted adult vaccination primarily toward high-risk groups (e.g., older adults and individuals with chronic conditions), potentially reinforcing the view that vaccination is not routinely indicated for healthy adults [28].

Together, these findings indicate that addressing vaccine hesitancy requires transparent communication of safety and efficacy data, regular updates from government agencies, and proactive engagement from trusted HCPs to build confidence and acceptance [29]. Given that willingness to vaccinate increased substantially with HCP recommendation, equipping HCPs to communicate safety and efficacy clearly will be key to alleviating concerns. Equally important is communicating the vaccine’s cost-effectiveness to the public, highlighting that it not only prevents illness but also reduces household and health system costs, reinforcing its value as an investment in health [30].

In comparison to the moderate scores in Knowledge and Attitudes, the low Practice score underscores a critical gap; theoretical knowledge and favourable attitudes do not translate into behaviour. Although Thailand offers multiple dengue prevention measures, including larvicide use, community cleanup and school-based education, individual uptake remains limited. For example, school-based programmes promote early awareness of source reduction, and community mobilisation initiatives such as “Every Friday Dengue-Free Day” encourage routine household clean-ups to eliminate breeding sites [31,32]. This pattern suggests that individuals may perceive limited benefit from broader prevention measures. To address this gap, interventions should remove behavioural and practical barriers, simplify actions and emphasise personal responsibility to complement public initiatives [33].

### 4.2. Factors Impacting Willingness to Vaccinate

To further encourage prevention behaviour, it is equally critical to identify and understand behavioural factors that influence willingness to vaccinate. WHO guidance emphasises that uptake improves when interventions address all components of Capability, Opportunity, and Motivation, and tailoring outreach accordingly ensures vaccination is both accessible and relevant to the target population [15,34].

A multivariable regression analysis using the COM-B framework identified a strong positive association between Reflective Motivation and willingness to vaccinate, underscoring the need for clear, credible information to support informed decisions. Messaging on vaccine safety, efficacy, and side effects, especially when delivered by trusted HCPs, government leaders and medical associations, can help address hesitancy. This is particularly important for those who are generally pro-vaccine but prefer to wait until safety is further assured. Prior evidence from Thailand shows that HCPs with strong digital or community presence can effectively shape public health behaviours [35].

Physical Opportunity was also positively associated with willingness, indicating that perceived ease of access plays a key role. This underscores the importance of ensuring that vaccines are available at familiar and convenient locations, such as public primary care clinics, schools, and workplaces. Evidence shows that TAK-003 can be coadministered with hepatitis, yellow fever and 9-valent human papillomavirus vaccines, provided different injection sites are used, and the WHO has recommended such coadministration in its guidance [36,37,38]. Given the observed link between influenza and dengue vaccine acceptance in Thailand, opportunities to explore coadministration with influenza and other routine vaccines may warrant further evaluation as a strategy to strengthen uptake. More broadly, integrating dengue vaccination into the national immunisation schedule alongside other adult and adolescent vaccines would maximise convenience and help reduce missed opportunities [39,40].

Lastly, greater household size was linked to higher willingness, possibly due to heightened perceived risk or a stronger sense of collective protection. Targeted outreach to larger or multi-generational households may therefore help strengthen vaccine confidence and coverage, as supported by evidence on home-based vaccination strategies in similar settings [41].

Conversely, a negative association between Physical Capability and willingness to vaccinate against dengue suggests that individuals who already engage in multiple preventive practices may perceive vaccination as less necessary. However, modelling evidence from a Thailand-based cost-effectiveness study found that vaccination could avert 41% of symptomatic dengue cases and 50% of hospitalised cases, offering substantial potential public health benefits alongside existing vector control measures [30]. This underscores the importance of communication strategies that convey vaccination’s added value even among individuals already engaging in preventive behaviours, particularly in reducing symptomatic and severe cases.

### 4.3. Stakeholder Collaboration and Phased Implementation to Design a Multi-Pronged Dengue Prevention Strategy

Based on the insights from this study, the Thai population prefer a comprehensive dengue prevention programme, whereby dengue vaccination, public education, vector control, and workplace health initiatives are integrated into a single strategy. There are several key considerations involving stakeholder collaboration and activities to ensure smooth and effective implementation.

#### 4.3.1. Stakeholder Collaboration

Successful programme design hinges on clearly defined roles, transparent coordination, and sustained engagement across stakeholder groups, which help prevent duplication of efforts, improve accountability, and enable adaptive responses to community needs [42]. National and local policymakers, such as the Department of Disease Control (DDC) and the Department of Health Service Support (DHSS), are central to setting strategic direction, aligning departments, and establishing enabling regulatory and financial frameworks. Local administrative organisations can further mobilise communities, particularly those in underserved areas, to support education and vaccination efforts. Empowering local and provincial authorities to implement vaccination as part of an integrated dengue control strategy, prioritising populations and geographic areas identified through dengue epidemiological data, will be critical to ensuring that vaccination is effectively embedded within broader prevention and vector control initiatives.

Village Health Volunteers (VHVs), as trusted local actors, are well-positioned to disseminate health messages, promote vaccine literacy, and assist with tasks such as registration and follow-up [33]. Public and private HCPs are equally crucial in delivering vaccines, addressing hesitancy, and reinforcing prevention during routine care. This emphasis on trusted local actors is supported by study findings, which show that willingness to vaccinate increased substantially with HCP recommendation, underscoring their importance in addressing hesitancy.

Academic institutions and professional societies can contribute by generating evidence to guide policy, monitoring programme outcomes, and conducting operational research on real-world vaccine performance. Meanwhile, the private sector can expand access through workplace-based initiatives and co-fund outreach campaigns, while international partners such as the WHO and the International Vaccine Institute (IVI) support technical capacity and share best practices. By coordinating across these diverse actors, Thailand can implement a responsive dengue control strategy with vaccination as a central pillar.

#### 4.3.2. Activities to Integrate Dengue Vaccination to Achieve a Multi-Pronged Dengue Management Programme

Effective stakeholder collaboration lays the foundation for a successful dengue vaccination programme, but phased integration is critical to ensure smooth rollout and national scale-up.

In the short term, the government should communicate its plans for an integrated dengue control strategy that positions vaccination as a key opportunity alongside existing prevention measures. Leveraging dengue surveillance data to identify high-burden areas and at-risk populations will be essential. These priority zones can serve as pilot sites for integrating vaccination into existing platforms such as workplace health programmes, school-based campaigns, and primary care clinics, which evidence shows can reduce access barriers, enhance convenience, and improve uptake in priority groups. These efforts will generate real-world data to inform national planning. Building on early insights, vaccination can then be gradually extended via sub-district health centres and embedded into routine care in high-risk areas.

Long-term dengue strategy should be guided by programmatic data on disease burden, vaccine coverage, safety, effectiveness, cost-effectiveness, and local endemicity. Provinces with persistently high transmission should be prioritised, particularly where populations are at increased risk of severe disease and death due to underlying comorbidities. Tailored delivery models, including school-based vaccination, should be a central focus, as children and young adults (ages 10–24) represent the highest incidence group in Thailand [4].

To ensure equitable access, expanded delivery points and strengthened cold chain capacity will be essential, particularly in underserved areas. Flexible models such as mobile clinics and community health workers have shown success in overcoming geographic barriers and improving trust and uptake [42]. Integration with existing schemes like the Social Security Scheme (SSS) and Civil Servant Medical Benefit Scheme (CSMBS) can help reduce out-of-pocket costs and improve vaccine accessibility. Ultimately, long-term efforts should be aligned with provincial health priorities, with national-level policy, financing, and communication reinforcing an integrated and decentralised approach.

This phased, evidence-driven strategy can enable effective integration of dengue vaccination into Thailand’s broader control efforts, improving long-term outcomes and system resilience.

### 4.4. Strengths and Limitations

This study provides valuable insights into the Thai population’s Knowledge, Attitudes, and Practices toward dengue prevention and vaccination, applying the COM-B framework to identify behavioural drivers of vaccine willingness. However, several limitations should be noted. Self-reported data remain prone to recall errors and social desirability bias, and the online format may have excluded less digitally connected groups, such as some older adults or rural residents. In addition, the use of a quota-based online consumer panel, with participation limited to adults aged 20–60 years with email and internet access, may limit external validity, as individuals who are not enrolled in online panels, outside the specified age range, or with limited internet access may be underrepresented. As a non-probability, online panel-based survey, the study is subject to selection and coverage bias, as individuals without reliable internet access or not enrolled in consumer research panels had no probability of selection. While sampling balanced age, gender, and region, factors such as education, prior dengue infection, and health literacy were not controlled for, potentially leaving residual confounding. Furthermore, the quota-based sampling approach introduced implicit stratification across multiple demographic subgroups, which informed how pooled and subgroup-level findings were interpreted. Accordingly, overall results are presented as descriptive summaries of the surveyed sample, while subgroup analyses and multivariable regression results are interpreted as indicative of associations and patterns within the study population rather than as precise population-level estimates. Future studies may build on this work by employing probability-based sampling designs and, where stratification is applied, ensuring that each stratum is adequately powered to support predefined inferential objectives. The regression findings, though informative, cannot imply causality given the cross-sectional design of the study. Additionally, contextual influences such as vaccine cost, cultural norms, or prior immunisation experiences were not examined in depth, but may influence vaccination uptake. Future studies using qualitative and quantitative methods and integrating non-self-reported data, such as observational assessments, could help validate these findings, offering a more comprehensive basis for targeted intervention strategies.

## 5. Conclusions

This study identifies important gaps between knowledge, attitudes, and preventive practices related to dengue among Thai adults. Although general awareness of dengue is moderate and willingness to vaccinate is relatively high, misconceptions around treatment, concerns about vaccine safety and cost, and low engagement in consistent preventive behaviours remain key barriers. Willingness to vaccinate is strongly influenced by motivation and perceived accessibility, highlighting the importance of trusted healthcare professionals and convenient delivery channels. Together, these findings underscore the need for clear, targeted communication and a multisectoral dengue prevention strategy that integrates vaccination with ongoing education and vector control efforts supported by coordinated government and healthcare stakeholder engagement, to strengthen sustainable dengue management in Thailand.

## Figures and Tables

**Table 1:** Sociodemographic characteristics of study respondents in Thailand.

Demographic	Sociodemographic	N (%), Total N = 600
Gender	Male	239 (40%)
Age	20–29 years old	142 (23.6%)
	30–39 years old	148 (24.7%)
	40–49 years old	148 (24.7%)
	50–60 years old	162 (27.0%)
Household size	1 member	29 (4.8%)
	2 members	92 (15.3%)
	3–4 members	327 (54.5%)
	5–6 members	125 (20.9%)
	>6 members	27 (4.5%)
Family household: children	No children	287 (47.8%)
	1 child	212 (35.3%)
	2 children	86 (14.3%)
	3 children	13 (2.2%)
	4 children	1 (0.2%)
	>4 children	1 (0.2%)
Ethnicity	Thai	585 (97.4%)
	Malay	1 (0.2%)
	Chinese	6 (1.0%)
	Thai-Indian	1 (0.2%)
	Others	7 (1.2%)
Religion	Buddhism	549 (91.5%)
	Christianity	14 (2.3%)
	Islam	27 (4.5%)
	Hinduism	2 (0.3%)
	Others	1 (0.2%)
	No religion	7 (1.2%)
Education level	Primary education	5 (0.8%)
	Secondary education	128 (21.3%)
	Tertiary education	427 (71.2%)
	Post-tertiary education	40 (6.7%)
Level of Income	Low: <25,000 THB	342 (57.0%)
	Medium: 25,000–30,000 THB	99 (16.5%)
	High: >30,000 THB	159 (26.5%)
Prior dengue infection	Yes	259 (43.2%)
Vaccinated against COVID-19	Yes	536 (89.3%)
Vaccinated against influenza	Yes	391 (65.2%)

**Table 2:** Prevention measures practised in Thailand.

Prevention Measures Practised	N (%), Total N = 600
Spray insect repellent/apply mosquito repellent patches	52%
Participate in community mosquito fogging	47%
Wear long-sleeved shirts and/or long pants	35%
Use wire mesh mosquito screens and/or mosquito nets	69%
Throw out any open bodies of water, e.g., plant containers.	73%
Perform periodic maintenance of water tanks	45%
Tightly cover all water containers	69%
Keep the drain free of blockage	48%
Place all garbage that can accumulate water in a closed bin	57%
Add larvicide in water containers to kill mosquito larvae	50%
Use an electric mosquito swatter	46%
Use guppy fish to consume a large no. of larval mosquitoes	47%
Apply citronella grass oil as a mosquito repellent	45%
Use chemical sprays (e.g., pyrethroid)	20%
Use mosquito coil or electric mosquito repellent	53%
None of the above	1%
Average no. of prevention measures conducted (out of 15)	7.6

**Table 3:** Individual willingness to vaccinate.

Demographic Characteristics	Sociodemographic Characteristics	Base	Mean	Standard Deviation	Willingness to Vaccinate Against Dengue (N, %)		
					High Willingness	Moderate Willingness	Low Willingness
Country/Region	Thailand	600	8.1	2.0	409 (68%)	176 (29%)	16 (3%)
	Global	3800	7.3	2.4	2014 (52%)	1482 (40%)	304 (8%)
	APAC	1400	6.8	2.2	574 (40%)	714 (52%)	112 (8%)
Gender	Male	239	8.2	1.9	168 (70%)	62 (26%)	9 (4%)
	Female	361	8.0	2.0	240 (66%)	114 (32%)	7 (2%)
Age	20–29 years old	142	8.1	1.7	94 (66%)	47 (33%)	1 (1%)
	30–39 years old	148	8.4	1.9	109 (74%)	37 (25%)	2 (1%)
	40–49 years old	148	7.9	2.1	96 (65%)	46 (31%)	6 (4%)
	50–60 years old	162	8.0	2.1	109 (67%)	46 (28%)	7 (4%)
Household Size	I live alone	29	6.7	2.4	11 (38%)	16 (55%)	2 (7%)
	2 members	92	8.0	2.0	62 (67%)	28 (30%)	2 (2%)
	3–4 members	327	8.1	1.9	219 (67%)	100 (31%)	8 (2%)
	5–6 members	125	8.4	1.9	94 (75%)	27 (22%)	4 (3%)
	>6 members	27	8.9	1.6	22 (82%)	5 (19%)	0 (0%)
Pregnant	Yes	25	8.2	1.5	18 (72%)	7 (28%)	0 (0%)
	No	334	8.0	2.0	222 (67%)	105 (32%)	7 (2%)
	I don’t know	2	6.0	1.4	0 (0%)	2 (100%)	0 (0%)
Education Level	No formal education	0	-	-	0 (0%)	0 (0%)	0 (0%)
	Primary education	5	6.6	3.0	2 (40%)	2 (40%)	1 (20%)
	Secondary education	128	8.1	2.0	87 (68%)	39 (30%)	2 (2%)
	Tertiary education	427	8.2	1.9	294 (69%)	122 (29%)	11 (3%)
	Post-tertiary education	40	7.5	2.3	25 (63%)	13 (33%)	2 (5%)
Region	Endemic	136	8.5	1.6	103 (76%)	32 (24%)	1 (1%)
	Non-endemic	464	8.0	2.0	305 (66%)	144 (31%)	15 (3%)
Level of Income	High	159	8.3	1.7	118 (74%)	38 (24%)	3 (2%)
	Medium	99	8.0	1.9	64 (65%)	34 (34%)	1 (1%)
	Low	342	8.0	2.1	226 (66%)	104 (30%)	12 (4%)
Prior Dengue Infection ^1^	Yes	259	8.3	1.8	190 (73%)	64 (25%)	5 (2%)
	No	341	7.9	2.1	218 (64%)	112 (33%)	11 (3%)
Vaccinated against COVID-19 ^2^	Yes	526	8.2	1.9	373 (70%)	154 (29%)	10 (2%)
	No	64	7.2	2.6	35 (55%)	22 (34%)	7 (11%)
Vaccinated against influenza ^2^	Yes	391	8.5	1.6	296 (76%)	91 (23%)	4 (1%)
	No	209	7.4	2.3	112 (54%)	85 (41%)	12 (6%)

^1^ Based on respondents’ perception, regardless of their actual dengue infection serostatus; ^2^ Based on respondents’ perception, regardless of their actual COVID or influenza vaccination status.

**Table 4:** Capability, Opportunity, and Motivation factors associated with willingness to vaccinate against dengue in Thailand.

	N = 600			
COM-B	Level (%)	Level (%)	Coefficient	*p*-Value
Capability—Physical	57%	50%	−1.48	<0.001
Capability—Psychological		59%	0.65	0.315
Opportunity—Physical	67%	78%	4.24	<0.001
Opportunity—Social		66%	−1.14	0.072
Motivation—Automatic	60%	38%	−0.21	0.372
Motivation—Reflective		60%	8.33	<0.001

## Data Availability

The original contributions presented in this study are included in the article. Further inquiries can be directed to the corresponding author.

## Supplementary Materials

The following supporting information can be downloaded at: https://www.mdpi.com/article/10.3390/ijerph23030290/s1, Table S1: Definition and derivation of Knowledge, Attitudes, and Practices sub-categories of KAP framework; Table S2: Definition and derivation of Capability, Opportunity, and Motivation sub-categories of COM-B framework; Table S3: Capability, Opportunity, and Motivation covariates used in multivariate regression; File S1: Thailand dengue KAP study screener and main survey.

## Abbreviations

The following abbreviations are used in this manuscript:

APAC	Asia-Pacific
CHERRIES	Checklist for Reporting Results of Internet E-Surveys
COM-B	Capability, Opportunity, Motivation–Behaviour behavioural model
COVID-19	Coronavirus Disease 2019
CSMBS	Civil Servant Medical Benefit Scheme
DDC	Department of Disease Control, Ministry of Public Health
DHSS	Department of Health Service Support, Ministry of Public Health
FDA	Food and Drug Administration
GEMKAP	Growth and Emerging Markets Knowledge, Attitudes and Practices
HCP	Healthcare Professional
IRB	Institutional Review Board
KAP	Knowledge, Attitudes, and Practices
THB	Thai Baht
VHV	Village Health Volunteer
